# Current issues in postmortem imaging of perinatal and forensic childhood deaths

**DOI:** 10.1007/s12024-016-9821-x

**Published:** 2017-01-12

**Authors:** Owen J. Arthurs, John C. Hutchinson, Neil J. Sebire

**Affiliations:** 1 Department of Radiology, Great Ormond Street Hospital for Children NHS Foundation Trust, London, WC1N 3JH UK; 2 Institute of Child Health, UCL, London, UK; 3 Department of Histopathology, Great Ormond Street Hospital for Children NHS Foundation Trust, London, UK

**Keywords:** Autopsy, Perinatal, Imaging, Forensic, Pediatric, MRI

## Abstract

Perinatal autopsy practice is undergoing a state of change with the introduction of evidence-based cross-sectional imaging, driven primarily by parental choice. In particular, the introduction of post mortem magnetic resonance imaging (PMMR) has helped to advance less-invasive perinatal autopsy in the United Kingdom (UK) and Europe. However, there are limitations to PMMR and other imaging techniques which need to be overcome, particularly with regard to imaging very small fetuses. Imaging is also now increasingly used to investigate particular deaths in childhood, such as suspected non-accidental injury (NAI) and sudden unexpected death in infancy (SUDI). Here we focus on current topical developments the field, with particular emphasis on the application of imaging to perinatal autopsy, and pediatric forensic deaths. Different imaging modalities and their relative advantages and disadvantages are discussed, together with other benefits of more advanced cross-sectional imaging which currently lie in the research domain. Whilst variations in local imaging service provision and need may determine different practice patterns, and access to machines and professionals with appropriate expertise and experience to correctly interpret the findings may limit current practices, we propose that gold standard perinatal and pediatric autopsy services would include complete PMMR imaging prior to autopsy, with PMCT in suspicious childhood deaths. This approach would provide maximal diagnostic yield to the pathologist, forensic investigator and most importantly, the parents.

## Introduction

Perinatal autopsy practice is undergoing a state of change with the introduction of evidence-based cross-sectional imaging, driven primarily by parental choice. In particular, the introduction of post mortem magnetic resonance imaging (PMMR) has helped to advance less-invasive perinatal autopsy in the UK. However, there are limitations to PMMR and other imaging techniques which need to be overcome, particularly with regard to imaging very small fetuses. Imaging is also now used to investigate particular deaths in childhood, such as suspected non-accidental injury (NAI) and sudden unexpected death in infancy (SUDI). Here we focus on current topical developments the field, with particular emphasis on the application of imaging to perinatal autopsy, and pediatric forensic deaths. Different imaging modalities and their relative advantages and disadvantages will be discussed, together with other benefits of more advanced cross-sectional imaging which currently lie in the research domain.

## The need for minimally invasive autopsy

Fetal and pediatric autopsy rates are at historically low levels, with overall acceptance rates now around 12–15% in the USA and UK respectively [1, 2]. This decline is largely due to reduced parental acceptance, including reluctance based on a range of factors including moral or religious grounds, not understanding the benefits, fear of disfigurement, and delay in funeral plans [3]. In parallel, non-invasive imaging techniques have been evaluated to establish their clinical diagnostic utility in this field, to determine whether a less-invasive approach might provide similar information whilst being more acceptable to parents. Less invasive autopsy (LIA) is based on post-mortem imaging followed by targeted tissue examination using a variety of techniques including endoscopic guidance or image-guided biopsy [4]. Since neither selected tissue biopsy nor endoscopic examination and sampling allow adequate anatomical information to be obtained, it is essential that comprehensive imaging of the whole body is carried out beforehand.

Over the last 10 years, several studies have now reported that post mortem magnetic resonance (PMMR) imaging, together with ancillary investigations including microbiology and placental examination where appropriate, has very high agreement with a conventional invasive autopsy, especially in fetal and perinatal cases. The largest prospective trial of PMMR versus standard traditional autopsy (Magnetic Resonance Imaging in Autopsy: MARIAS study) reported >90% concordance in fetuses and stillbirths, and 75% concordance in children [5]. Published evidence suggests that this is an acceptable approach for the majority of clinical staff [6] and parents [7, 8] alike. In some cases, parents may not agree to any form of autopsy tissue sampling but post-mortem imaging combined with ancillary investigations, such as placental histological examination, can still provide useful clinical information.

## Recent advances in post mortem imaging

**Fig. 1:**
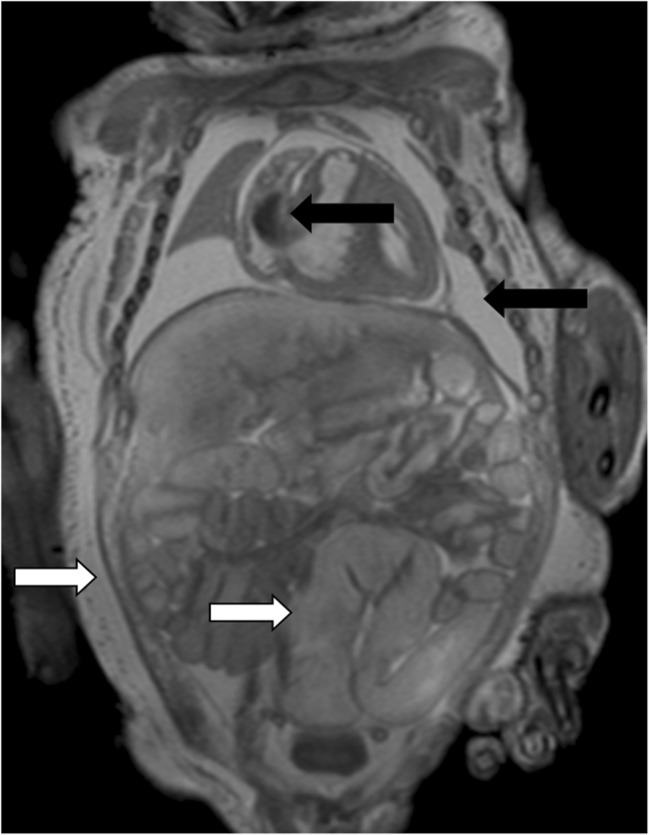
Normal appearances on PMMR. Coronal T_2_-weighted PMMR in a late gestation stillbirth, demonstrating normal physiological post mortem imaging appearances. There is intracardiac gas, pleural and pericardial effusions in the chest (*black arrows*), bowel dilatation and widespread subcutaneous edema (*white arrows*), all of which can be misinterpreted as pathological to radiologists unfamiliar with autopsy imaging

**Fig. 2:**
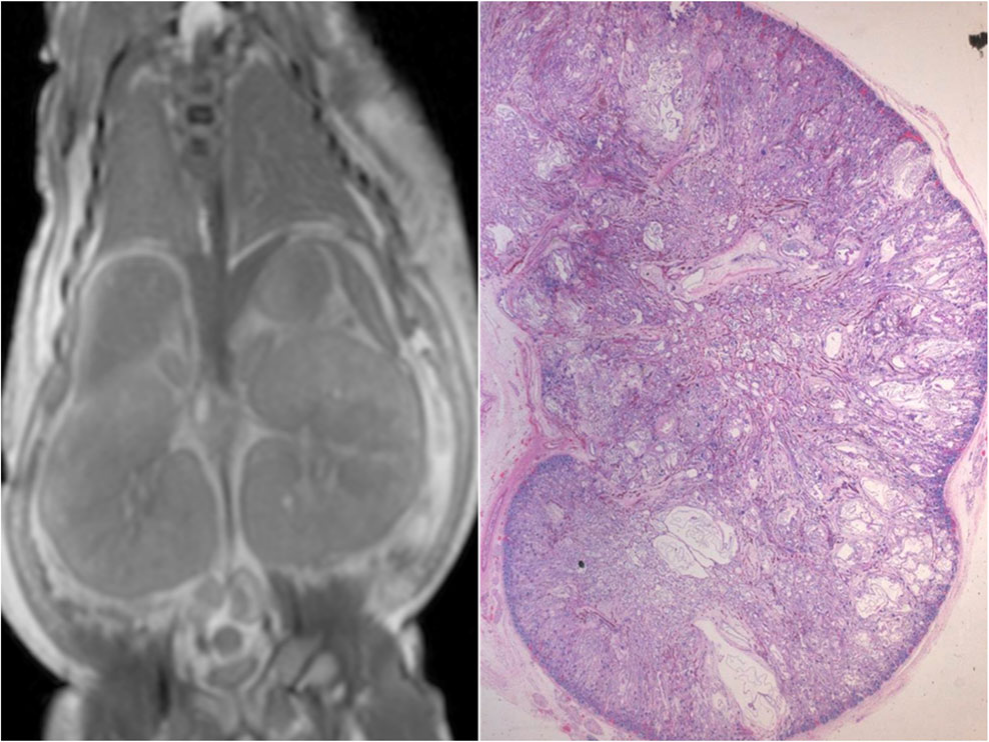
PMMR of congenital abnormalities. PMMR is particularly good for congenital anatomical abnormalities, such as intracranial hemorrhage, brain malformations, renal anomalies, congenital heart disease and skeletal dysplasias. This example shows bilateral enlarged high signal kidneys at coronal T_2_-weighted PMMR in a 27 week gestation fetus, which are classical features of autosomal recessive polycystic kidney disease (**a**), confirmed at microscopy (**b**)

Not only has the diagnostic accuracy and diagnostic acceptability of PMMR now been demonstrated, but limitations of the technique have also been documented. Whilst PMMR has been validated for several other activities usually performed during autopsy, such as organ weight or volume estimation [9, 10], there is a steep learning curve in PMMR acquisition and reporting, as would be expected with any new imaging development. In particular, recognizing normal PM changes which occur such as fluid redistribution (subcutaneous edema, pleural and pericardial effusions and ascites) can be challenging to radiologists unfamiliar with autopsy work [11, 12] (Fig. 1). As would be expected, PMMR is particularly good for congenital anatomical abnormalities, such as intracranial hemorrhage, brain malformations, renal anomalies (Fig. 2), congenital heart disease and skeletal dysplasias [13–16]. However, several normal changes may be misinterpreted for disease states, such as bowel dilatation as obstruction [17] or normal pulmonary changes as pneumonia [11, 14]. Conventional PMMR is particularly poor at detecting microscopic changes such as renal dysplasias and disseminated sepsis, which often have no imaging correlate [14, 17].

**Fig. 3:**
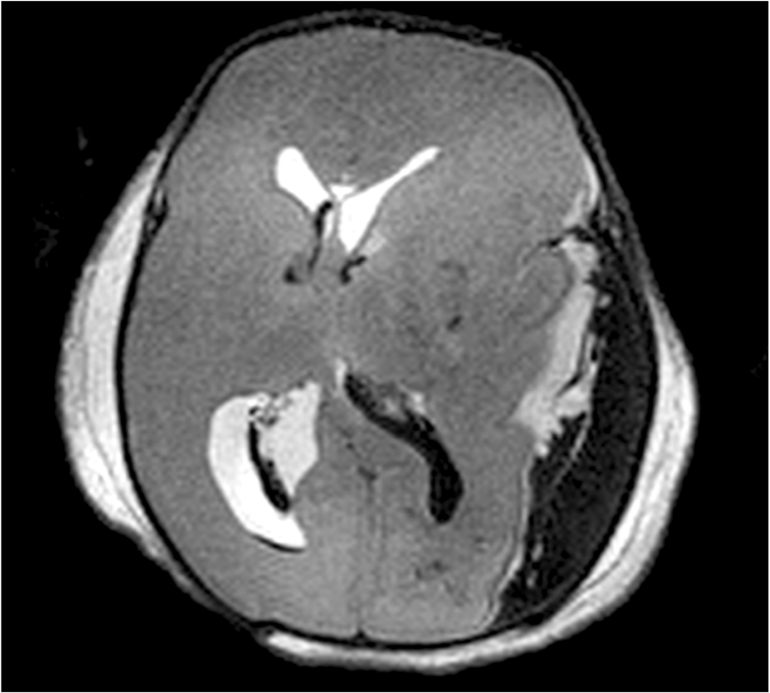
PMMR to document injuries. Post mortem imaging is useful to document the extent of intracranial injury prior to autopsy. In this case, axial T2-weighted PMMR demonstrates a large left parietooccipital subdural hemorrhage, which is causing mass effect on the brain. There is a small amount of intraventricular hemorrhage in addition

In imaging childhood deaths, PMMR can be particularly useful to delineate traumatic injuries prior to autopsy, including any internal hemorrhage, visceral or mesenteric injury associated with blunt or penetrating trauma to the body. It can give precise details about soft tissue injuries associated with rib fractures, the severity of intracranial injury (e.g. Fig. 3), and soft tissue limb injury. PMMR can be useful to show complications from inaccurate intraosseous needle placements, including unilateral soft tissue edema or gas tracking up the lower limbs [18].

**Fig. 4:**
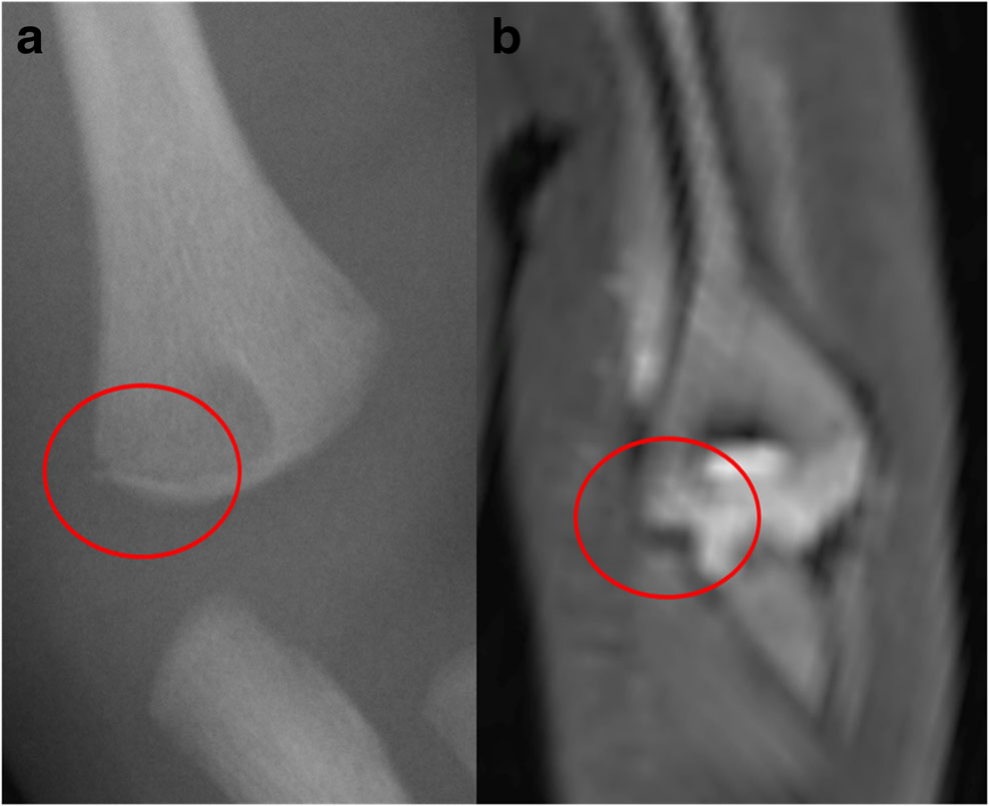
Fracture detection on PMMR. Long bone fractures are still probably best imaged using conventional radiographs, as these demonstrate long bone fractures adequately and some subtle fractures may be missed on PMMR, such as this corner metaphyseal fracture of the left distal humerus in a 5 month old child. It was identified on conventional radiography (**a**) but deemed too subtle to be identified on PMMR (**b**). Reproduced with permission from [16]

Bone fractures are still probably best imaged using conventional radiographs, as these demonstrate long bone fractures adequately and some subtle fractures may be missed on PMMR [16] (Fig. 4). The precise contribution that PMMR makes to a forensic autopsy will be on a case-by-case basis, but any additional information regarding the extent of intracranial injury, extent of thoracic and abdominal injury, number and site of fractures, can be useful to guide the forensic pathologist in conducting their detailed examination (Fig. 3).

## Post mortem CT or MRI?

**Fig. 5:**
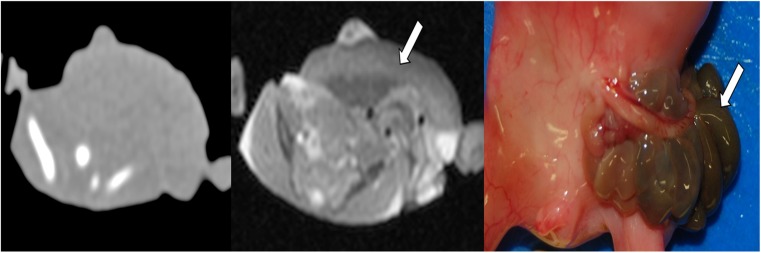
Example of non diagnostic PMCT. PMCT has several disadvantages, including reduced soft tissue contrast due to reduced abdominal and subcutaneous fat. In an 18 week fetuses, an abdominal wall defect was clearly diagnosed on PMMR (**b**), with liver and small bowel loops herniated outside of the normal abdominal cavity in gastroschisis (*white arrow*), but the PMCT in the same patient was non-diagnostic. Gastroschisis was clearly identified at autopsy (**c**; *white arrow*). Reproduced with permission from [22]

Post mortem computed tomography (PMCT) is becoming widely used in the adult autopsy setting, particularly with the addition of vascular contrast medium to create PMCT angiography [19, 20]. The main advantages of PMCT over PMMR are speed of acquisition, availability in most hospitals, and the better bone detail that is achieved using unenhanced CT. However, in children, PMCT has several disadvantages, including reduced soft tissue contrast due to reduced abdominal and subcutaneous fat, poor soft tissue contrast in the brain, and without vascular contrast medium, assessment of the thoracic and abdominal cavity is particularly challenging [21] (Fig. 5).

Recently, unenhanced PMCT has been shown to perform worse than PMMR in children. Preliminary studies of PMCT in infants showed 90% accuracy in a large proportion of deaths which remained unexplained [22], but the main problem with PMCT is that it is largely non-diagnostic in the smaller bodies which are often referred for perinatal autopsy [22]. PMMR performs better than PMCT in the same individuals, largely because of PMCT’s relatively high non-diagnostic rates [22]. Without the addition of intravenous contrast (via femoral, umbilical vessels or direct intracardiac injection) for angiography [23, 24], or ventilating the lungs to improve lung imaging [25], PMCT typically performs worse than PMMR apart from for bone imaging (e.g. rib fractures), which is discussed later in this article in more detail.

**Fig. 6:**
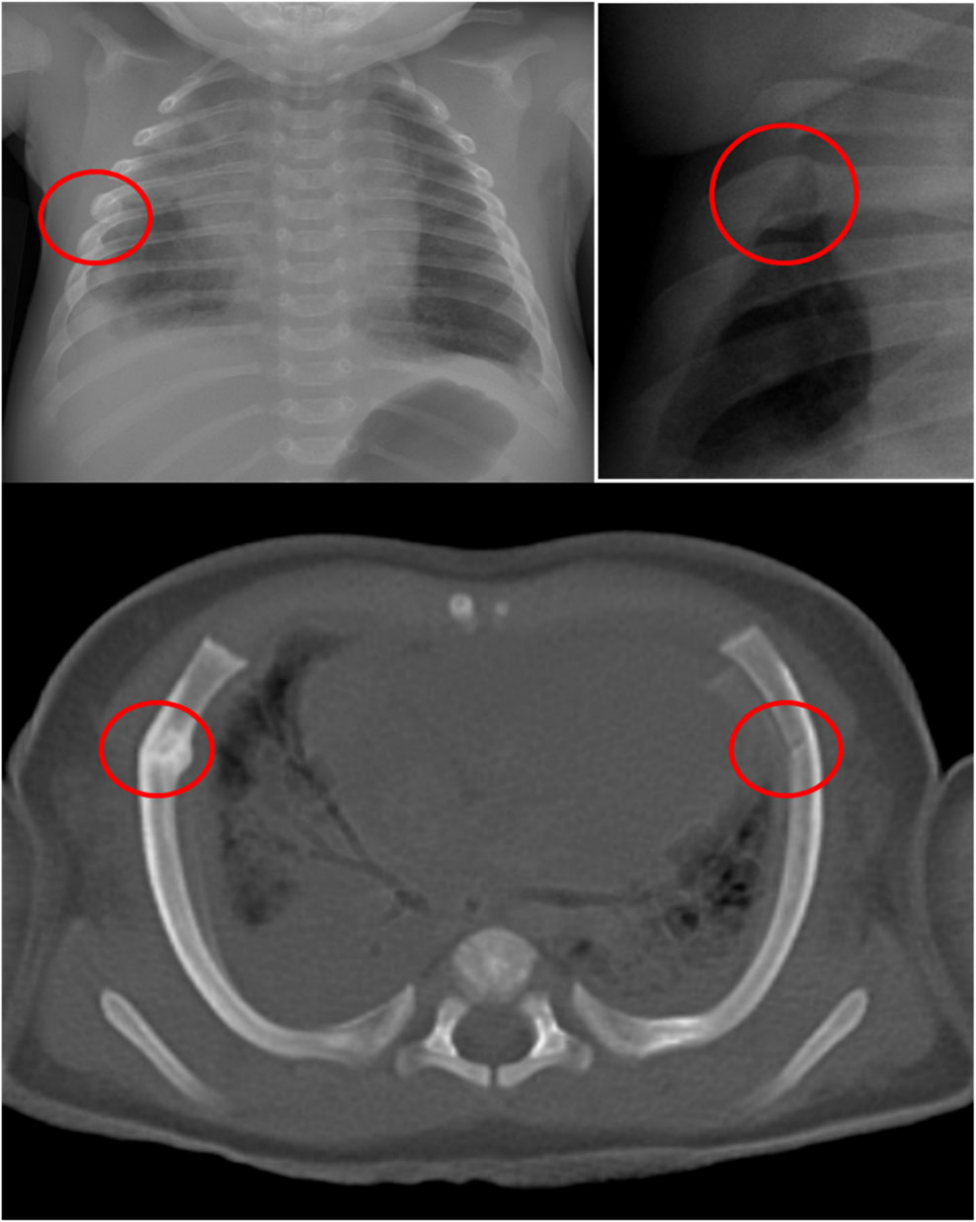
PMCT for fractures. PMCT is becoming particularly useful at detecting rib fractures. An example is given of a 4 month old girl with a rib fracture on the right, difficult to identify on the frontal chest radiograph (**a**), easier to see on the oblique view of the left sided ribs (**b**), and very easy to identify on axial 3D reconstructed PMCT (**c**). PMCT confirms that there are bilateral anterolateral fractures, in a typical location resuscitation related injuries, and there were no other signs of injuries in this child. Fresh anterolateral fractures are highly likely to be related to resuscitation if there are no other associated injuries

PMCT is becoming particularly useful at detecting rib fractures, and detailed abnormalities of other bone injuries (Fig. 6). As both PMCT and PMMR become more widely used in perinatal autopsy and the forensic setting, their diagnostic utility will become established as both radiologists and pathologists gain experience.

## Sudden unexpected death in infancy (SUDI)

In 2013, there were 2686 infant deaths in England and Wales [26]; of these, approximately 1000 will have presented as a sudden, unexpected death (SUDI). Due to the nature of these cases, they usually require investigation through HM Coroner (in England, Wales and Northern Ireland) or the Procurator Fiscal (Scotland). SUDI presentations represent the commonest group of infant deaths undergoing autopsy examination [27] but despite a relatively high throughput of cases, SUDI remains poorly understood, and investigation of SUDI deaths are a challenging area for medical practitioners. The majority of investigations within the Coronial / Fiscal system require a cause of death to be established to a standard of proof equating to ‘on the balance of probability’, rather than ‘beyond reasonable doubt / so as to be sure’. Even so, the majority of SUDI deaths remain unexplained [28]. Of the cases where a cause of death may be identified, the potential underlying may be subtle, thus requiring a rigorous approach to the investigation. A keen understanding of the rapid physiological changes that occur during the first year of life is therefore essential when considering SUDI cases. The investigative strategy recommended by the Royal College of Pathologists is extensive [29] but largely based on expert opinion, rather than evidence. An updated guideline is expected to be published in 2016. A recent systematic review evaluating SUDI investigation strategies described the need for mandatory investigation of SUDI cases, ideally through specialist centers, that can provide strong leadership and integrate with Coronial services [30].

Some presentations of SUDI will be attributable to natural causes (undiagnosed infections, congenital malformations), whilst some will occur in unnatural circumstances, including accidents, and some within the context of inflicted injury [28]. Of the SUDI cases that remain unexplained, some will occur in association with well-described risk-factors, such as co-sleeping, prone sleeping, soft-bedding, parental smoking or drug use, and socio-economic deprivation [30–33] though a definitive cause of death may elude the investigative team. A proportion of unexplained SUDI cases may be classified as SIDS (Sudden Infant Death Syndrome) if the cause and mechanism of death cannot be explained following completion of all investigations and the death occurred during normal sleep. SIDS remains a diagnosis of exclusion, and in practical terms is likely to represent a heterogeneous mixture of cases with complex, multifactorial causes that current gold standard investigations cannot adequately detect or classify [26, 34].

It is relatively rare to detect a cause of death macroscopically at a SUDI autopsy [28]. The macroscopic autopsy procedure is undertaken to exclude traumatic injuries and obtain samples for microbiological, biochemical, toxicological and genetic analysis that may identify a definitive cause of death. Post-mortem imaging can therefore assist in several ways; firstly, cross-sectional imaging can identify cases with a structural lesion present. Secondly, imaging can then be used live within the mortuary to facilitate a targeted procedure (e.g. localized infection identified on PMMRI sampled for histology and microbiology under ultrasound guided biopsy). Such approaches are increasingly popular with Coroners, as targeted investigation permits a cause of death to be identified in a timely manner whilst minimizing distress associated with the autopsy. This is particularly true for Muslim and Jewish families, for many of whom the idea of an autopsy remains ideologically unacceptable [35]. Thirdly, negative findings obtained by imaging may be of importance to families and clinicians if there is there are specific questions surrounding the death, for example, in the context of a family history of congenital heart disease. As “omic” approaches to diagnosis continue to expand, tissue sampling is likely to further increase in importance in the context of SUDI deaths, with next-generation sequencing and proteomic studies showing promise within this field [36–40]. As a result, imaging techniques, including laparoscopically assisted biopsy and ultrasound-guided biopsy, are likely to become essential skills for pathologists and radiologists involved in post-mortem investigations in order to obtain tissue for diagnostic and research purposes. Furthermore, given the difficulties in establishing asphyxia as a cause of death in this group, it is possible that future developments, such a MR spectroscopy evaluation, may provide additional specific information regarding mechanisms and timing of death [41]. This is of particular importance given the recognition of the increasing relative frequency of co-sleeping associated infant deaths, and their mechanisms.

## Non-accidental death

Comprehensive skeletal radiography is a well-established part of the routine investigation of suspicious childhood deaths, particularly with regard to suspected non-accidental injury (NAI). Skeletal radiography provides an overview of bone structure and development, bone biometry and any specific bone abnormalities [42]. Detailed recommendations for performing and reporting skeletal imaging in suspected NAI are available from both Royal College of Radiologists (UK) [43], and American equivalent ACR [44]. Skeletal surveys are used in the NAI setting in order to identify, or exclude, occult or hidden fractures. Skeletal surveys have a high yield of revealing abuse, particularly in children under the age of 2 years, who may otherwise have no external manifestations. Typical occult fractures may involve corner metaphyseal injuries or posterior rib fractures, particularly in states of healing, which have a high specificity for non-accidental injury [45].

Whilst CT of the brain is widely used in suspected NAI, evidence from post mortem CT studies suggests that PMCT may also give a higher yield of fracture diagnosis in suspected NAI than conventional radiographs, particularly with regard to rib fractures. Acute, un-displaced rib fractures can be difficult to detect on radiography, particularly in the absence of any callus formation. Rib fractures following cardiopulmonary resuscitation are reportedly rare on skeletal radiographs, and when they do occur, are often multiple, anterolateral, incomplete (greenstick) fractures [45–47]. Fresh anterolateral fractures are highly likely to be related to resuscitation if there are no other associated injuries (Fig. 6) [46, 47]. One retrospective study showed that approximately twice as many fractures are identified on PMCT than radiographs, particularly subtle rib fractures [48], although rib fracture detection rates using both techniques are dependent on observer experience [48, 49]. PMCT may be particularly useful for fractures near the manubrium and sternum, and near the costovertebral junctions, which are particularly difficult to assess on radiographs. In the post mortem setting, there is little disadvantage to performing more detailed cross-sectional imaging such as PMCT, and an argument could be made that any imaging modality that could possibly increase the detection of injuries should be employed. Routine use of PMCT in non-accidental injury in live patients remains to be evaluated.

**Fig. 7:**
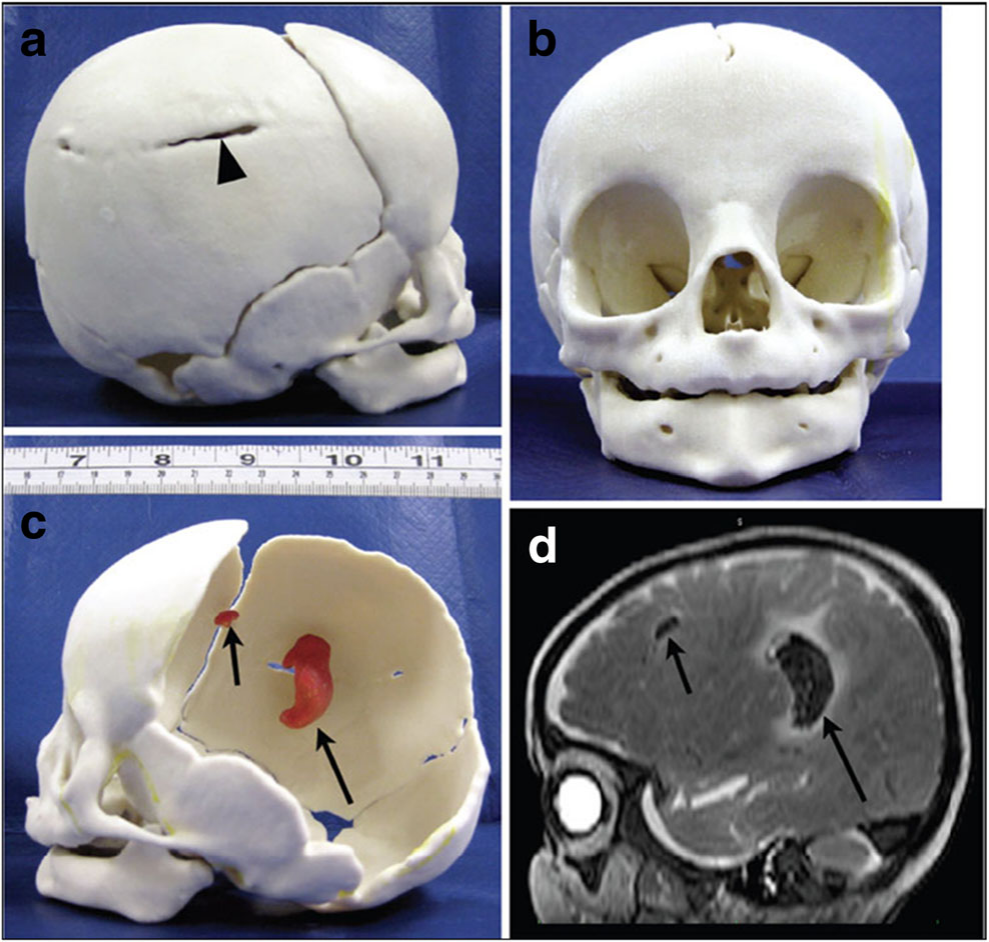
3D prototype printing. 3D model of a fracture skull and underlying brain hemorrhage in an infant brain. The PMCT dataset (to provide the 3D skull structure) was co-registered with the PMMR image (to identify the bleed volume and position) to give a composite image (**d**). This was printed into a skull (**b**), to demonstrate the fracture (*black arrow head*, **a**) and internal hemorrhage (*black arrows*, **c**). These findings were confirmed at autopsy. Reproduced with permission from [50]

There are other advantages of PMCT over conventional radiographs, above and beyond potential increased diagnostic yield. A permanent 3D record of detailed anatomical features can be stored in perpetuity, for teaching and training purposes, which is not achievable by current histopathological dissection. Detailed 3D imaging also lends itself to 3D models, which can be printed out and held in one’s hand (Fig. 7). Simple magnification allows larger replication of any pathological features, making it easier to appreciate normal and abnormal anatomy, and printed models can also be annotated and stored long-term for teaching purposes, improving understanding of 3D relationships of complex congenital abnormalities [50]. Clinicians may find these models particularly useful for explaining abnormalities to parents, as a “clean” model of the abnormalities without any autopsy photographs to be used. Some parents may wish to keep an artificial replica of the baby or organ, to help with the bereavement process. In medico-legal cases, juries and judges may benefit from being shown rapid prototyping model of the significant findings, rather than relying on photographs or drawings [50].

## Current research in post mortem imaging

The more imaging that is performed, the more other advantages of imaging become apparent aside from primary diagnosis. For example, in abandoned babies found dead, two primary questions are raised: was the baby born alive and subsequently died, or born dead (stillbirth), and can we determine the time interval since death? There is preliminary evidence that PMMR may be able to address both of these questions.

**Fig. 8:**
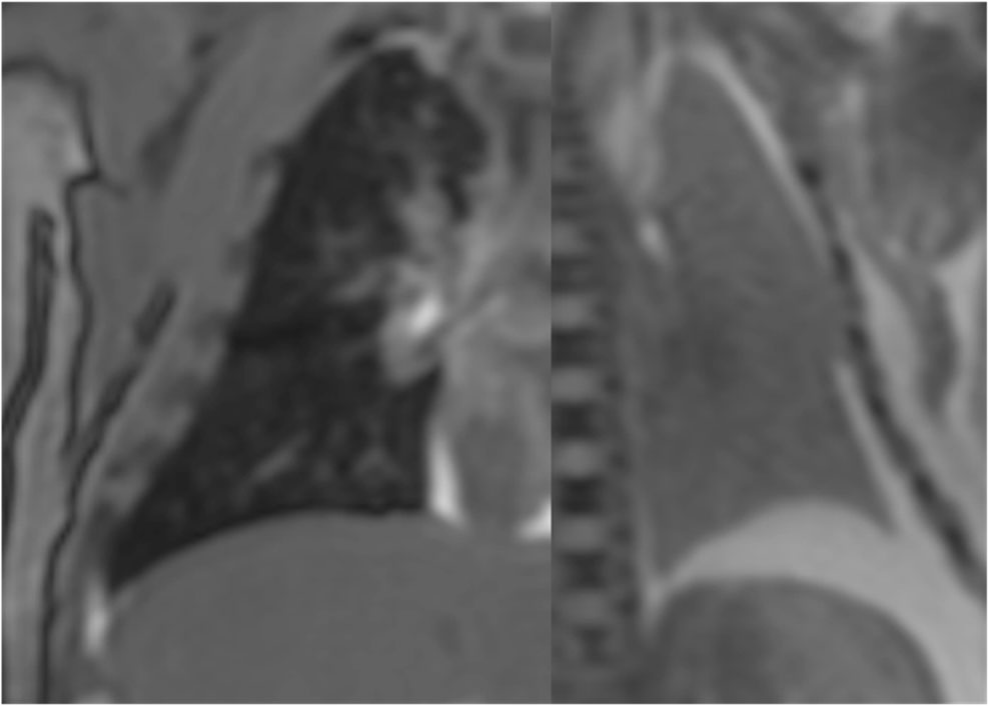
PMMR for lung aeration. Signal intensity differences in the lungs on PMMR may be used to differentiate between a baby who has breathed (dark airways and lungs on coronal T2-weighted PMMR image in a 2 week old baby; **a**) versus one that has not (light lungs in a 30 week gestation fetus with no signs of life at delivery; **b**). Reproduced with permission from [54] under Open Access agreement

Conventional autopsy techniques of establishing lung aeration include the lung flotation technique, an invasive test requiring lung evisceration and observing whether they float or sink when placed into water; floating lungs are traditionally deemed to contain air, suggesting breathing before neonatal demise [51], with published accuracy ranging from 37 to 95% [52, 53]. We have recently shown in a small group of subjects that subjective lung parenchyma aeration on PMMR is a good indicator of spontaneous breathing, with similar accuracy to lung flotation methods. This has the advantage of being completely non-invasive, may be acquired along with routine PMMR imaging, and may be useful to distinguish livebirth from stillbirth in most cases (Fig. 8) [54].

**Fig. 9:**
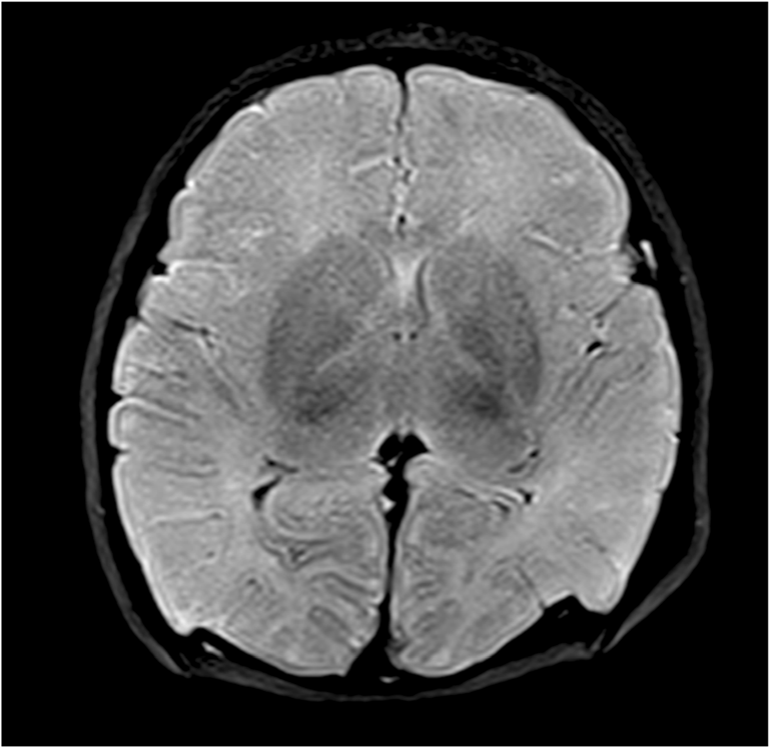
PMMR of hypoxic brain changes. Axial T2-weighted PMMR image through a fetal post mortem brain, showing an example of typical low signal change in the basal ganglia which may be associated with hypoxia. Conventional PMMR cannot currently distinguish antemortem from postmortem hypoxic change

Post mortem interval is more difficult to determine. Since some aspects of PMMR may be quantifiable, there is preliminary research to show that changes in imaging parameters may relate to the post mortem interval (time interval between death and imaging). For example, the accumulation of fluid in the lungs and pleural space following death results in signal changes on PMMR chest imaging. The rate of pleural fluid accumulation may correspond with post mortem interval in children [55], and the rate at which lung parenchymal air is replaced with fluid may also show correlations with post mortem interval [56]. Other studies have attempted to evaluate brain signal changes (e.g. hypoxic changes in the basal ganglia) with respect to post mortem interval (Fig. 9) [57]. It may be that a combination of quantifiable imaging parameters are needed to retrospectively estimate the time of death.

## Imaging in small fetuses

**Fig. 10:**
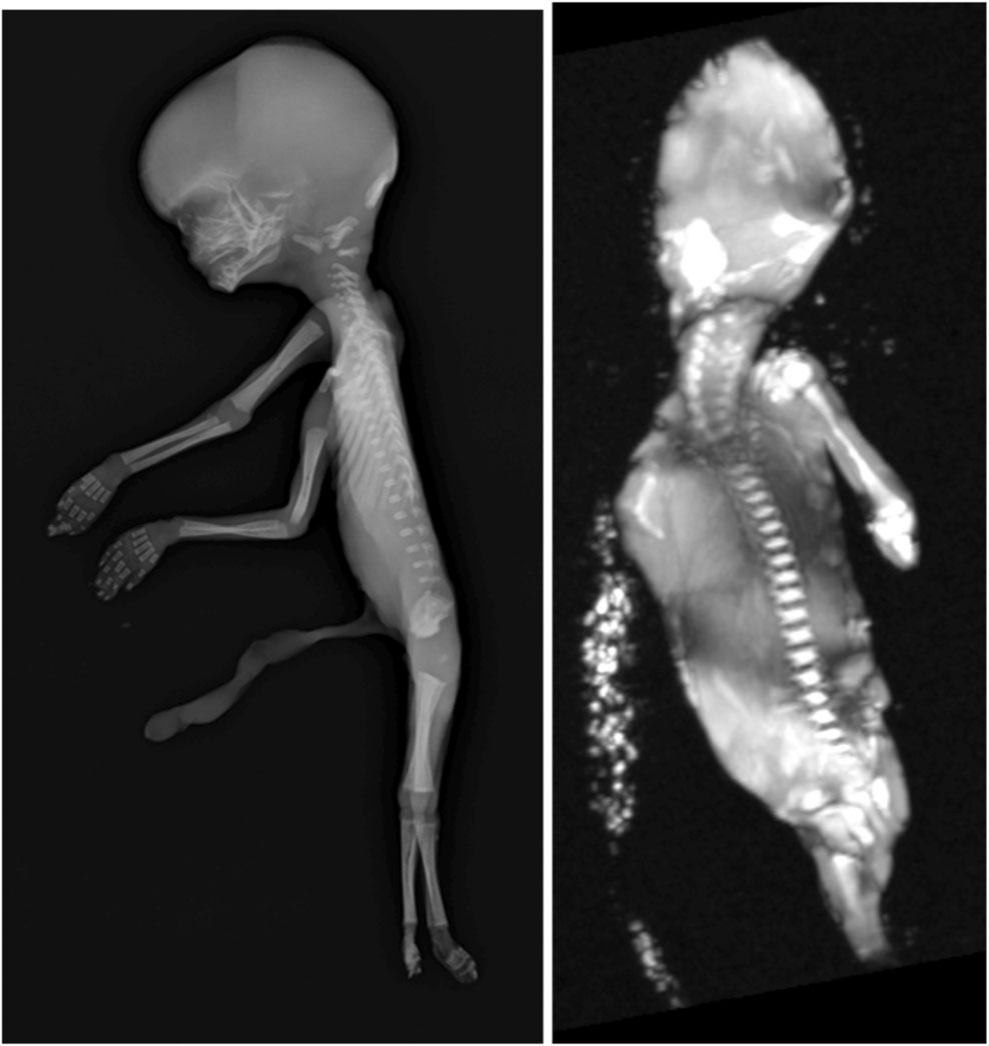
Limits of body PMMR imaging. 3D reconstruction from high resolution CISS PMMR image of a 14 week gestation (**b**). Skeletal radiography (**a**) at this gestation is often better than the highest resolution imaging at 1.5 T PMMR (**b**), which at low body weights is often non-diagnostic [58]

With the increasing introduction of routine first trimester antenatal US screening, fetuses are being submitted for autopsy examination at early gestational ages. These represent a challenge, not only for traditional autopsy examination but also post mortem imaging. Standard clinical PMMR at 1.5 T is non-diagnostic in around a third of <24 week gestation fetuses [5], and diagnostic performance drops significantly below 500 g bodyweight (Fig. 10) [58]; PMCT performs equally badly [22].

Techniques for post mortem imaging of very small and early gestation fetuses now includes PMMR at stronger field strengths, and micro CT. The diagnostic feasibility of very high field (9.4 T) PMMR has been described [59] although this technology is expensive, and time consuming and not widely available, although a case could be made for centralizing services for precisely this provision. 3 Tesla PMMR may become more widely available, and has been shown to have good diagnostic yield in particular for congenital cardiac abnormalities [60] but whether there is a significant diagnostic improvement over 1.5 T PMMR remains to be established.

**Fig. 11:**
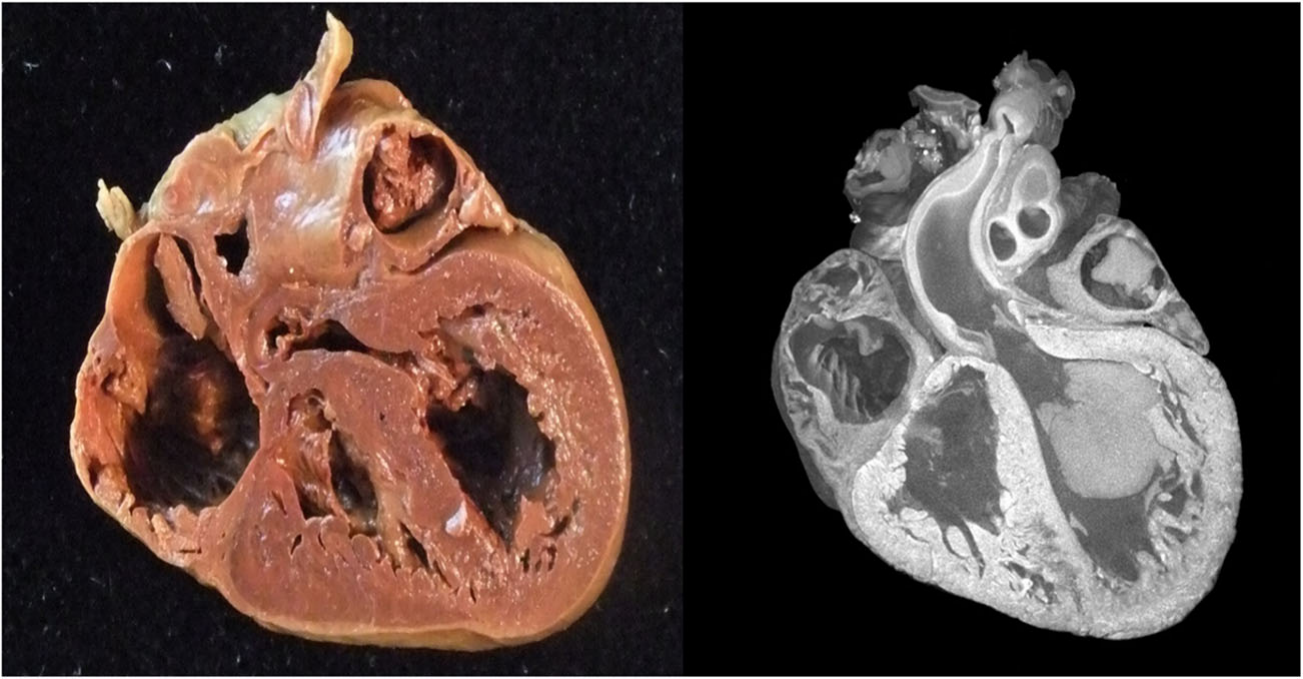
Micro CT of fetal heart. Normal fetal heart from an unexplained intrauterine fetal death at 23 weeks gestation (heart weight 5.3 g) at autopsy (**a**) with the corresponding micro-CT volume rendering at approximately 20 μm spatial resolution, following immersion in iodine (**b**). Adapted with permission from [62]

Micro-CT is another potential alternative diagnostic modality for imaging small objects, using CT technology but at improved resolution down to micrometers rather than millimeters. Micro-CT is described in post mortem forensic work [61] and has recently been used to show very high quality imaging with good diagnostic accuracy for fetal hearts (Fig. 11) [62, 63] and kidneys [64] although extracting and ‘fixing’ tissue for optimal contrast is necessary. Pilot studies of whole-body imaging using microCT are also promising [65]. Several of these techniques may improve PM imaging at low bodyweights where conventional PMMR is challenging [56] and virtual 3D datasets allow virtual dissection and re-dissection of organs, providing detailed examination and review without need for organ retention.

## Conclusion

The addition of post mortem imaging to conventional autopsy methods allows for autopsies to be offered to those who would ordinarily refuse, for detailed examination of the body prior to incisions as an adjunct to pathologists, to create 3D datasets for storage and teaching, and improved diagnostic accuracy compared to conventional techniques in certain settings. Whilst variations in local imaging service provision and need may determine specific practice patterns, including access to machines and appropriately skilled and experienced interpreters, the gold standard perinatal and pediatric autopsy service would include complete PMMR imaging prior to autopsy, with PMCT in suspicious childhood deaths. This would allow maximal diagnostic yield to the pathologist, forensic investigators and most importantly, the parents.

## Key points

1. Post mortem imaging techniques in children and fetuses have high concordance rates with traditional autopsy and show good levels of acceptability with parents.
2. PMCT is becoming useful for bony injuries, particularly the assessment of rib fractures.
3. PMMR can delineate traumatic injuries prior to autopsy, particularly precise details about soft tissue injuries associated with rib fractures, the severity of intracranial injury and soft tissue limb injury.
4. PMMR imaging may be useful to distinguish livebirth from stillbirth by assessment of lung aeration.
5. A gold standard perinatal and pediatric autopsy service would include complete PMMR imaging prior to autopsy, with PMCT in suspicious childhood deaths.
